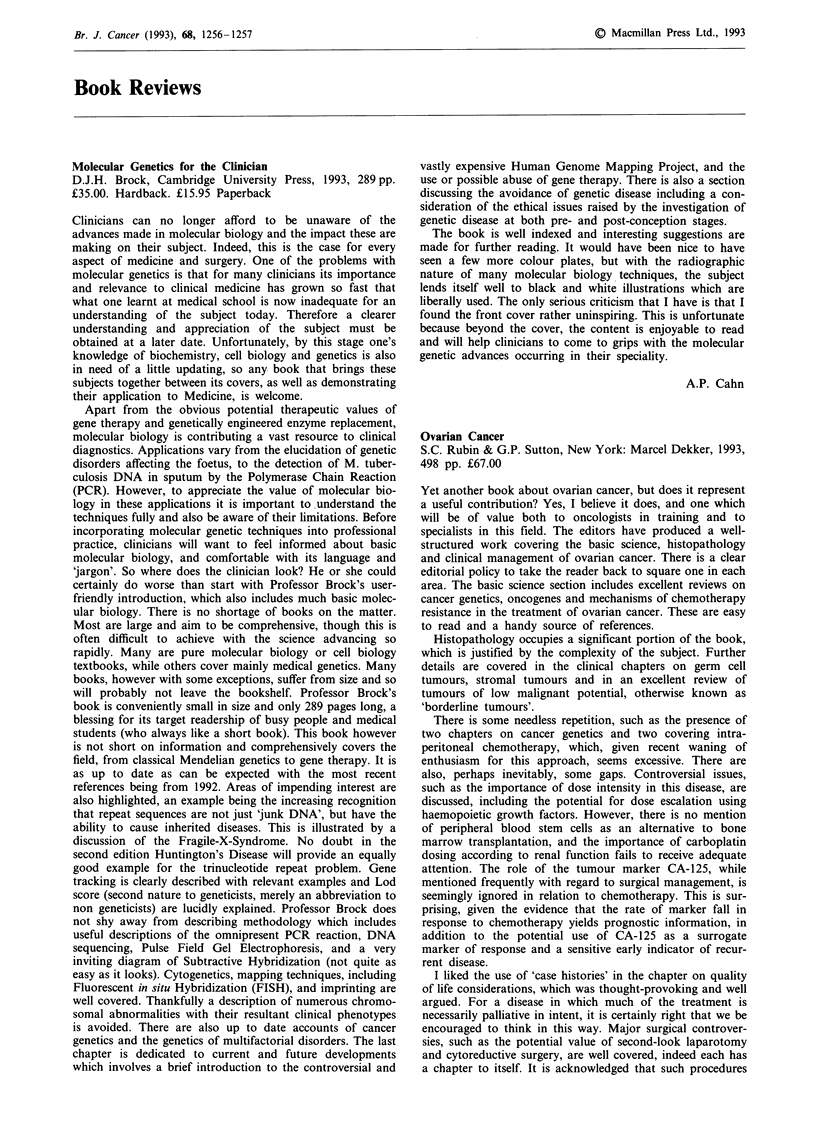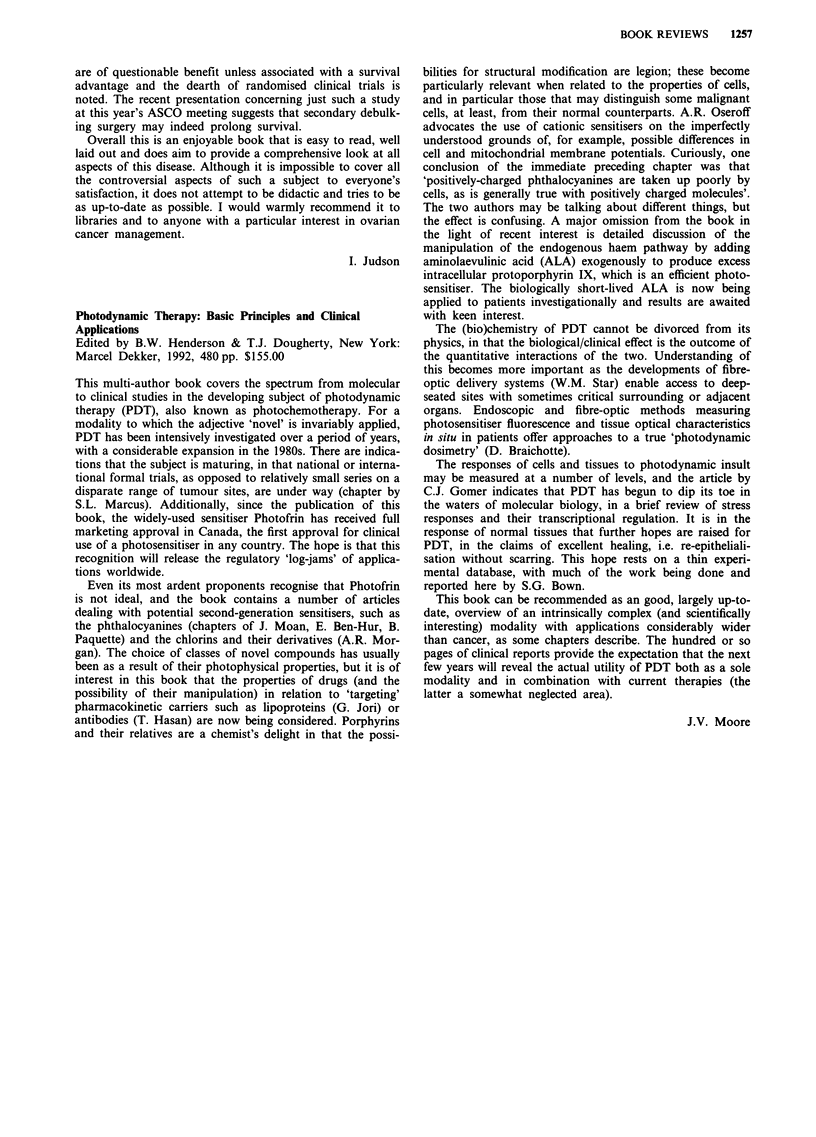# Ovarian Cancer

**Published:** 1993-12

**Authors:** I. Judson


**Ovarian Cancer**
S.C. Rubin & G.P. Sutton, New York: Marcel Dekker, 1993, 498 pp. £67.00

Yet another book about ovarian cancer, but does it represent a useful contribution? Yes, I believe it does, and one which will be of value both to oncologists in training and to specialists in this field. The editors have produced a well-structured work covering the basic science, histopathology and clinical management of ovarian cancer. There is a clear editorial policy to take the reader back to square one in each area. The basic science section includes excellent reviews on cancer genetics, oncogenes and mechanisms of chemotherapy resistance in the treatment of ovarian cancer. These are easy to read and a handy source of references.

Histopathology occupies a significant portion of the book, which is justified by the complexity of the subject. Further details are covered in the clinical chapters on germ cell tumours, stromal tumours and in an excellent review of tumours of low malignant potential, otherwise known as 'borderline tumours'.

There is some needless repetition, such as the presence of two chapters on cancer genetics and two covering intraperitoneal chemotherapy, which, given recent waning of enthusiasm for this approach, seems excessive. There are also, perhaps inevitably, some gaps. Controversial issues, such as the importance of dose intensity in this disease, are discussed, including the potential for dose escalation using haemopoietic growth factors. However, there is no mention of peripheral blood stem cells as an alternative to bone marrow transplantation, and the importance of carboplatin dosing according to renal function fails to receive adequate attention. The role of the tumour marker CA-125, while mentioned frequently with regard to surgical management, is seemingly ignored in relation to chemotherapy. This is surprising, given the evidence that the rate of marker fall in response to chemotherapy yields prognostic information, in addition to the potential use of CA-125 as a surrogate marker of response and a sensitive early indicator of recurrent disease.

I liked the use of 'case histories' in the chapter on quality of life considerations, which was thought-provoking and well argued. For a disease in which much of the treatment is necessarily palliative in intent, it is certainly right that we be encouraged to think in this way. Major surgical controversies, such as the potential value of second-look laparotomy and cytoreductive surgery, are well covered, indeed each has a chapter to itself. It is acknowledged that such procedures are of questionable benefit unless associated with a survival advantage and the dearth of randomised clinical trials is noted. The recent presentation concerning just such a study at this year's ASCO meeting suggests that secondary debulking surgery may indeed prolong survival.

Overall this is an enjoyable book that is easy to read, well laid out and does aim to provide a comprehensive look at all aspects of this disease. Although it is impossible to cover all the controversial aspects of such a subject to everyone's satisfaction, it does not attempt to be didactic and tries to be as up-to-date as possible. I would warmly recommend it to libraries and to anyone with a particular interest in ovarian cancer management.

I. Judson